# Initiation of esophageal squamous cell carcinoma (ESCC) in a murine 4-nitroquinoline-1-oxide and alcohol carcinogenesis model

**DOI:** 10.18632/oncotarget.3339

**Published:** 2015-01-21

**Authors:** Kwame Osei-Sarfo, Alison M. Urvalek, Xiao-Han Tang, Theresa Scognamiglio, Lorraine J. Gudas

**Affiliations:** ^1^ Department of Pharmacology, Weill Cornell Medical College, New York, USA; ^2^ Department of Pathology, Weill Cornell Medical College, New York, USA; ^3^ The Meyer Cancer Center, Weill Cornell Medical College, New York, USA

**Keywords:** 4-nitroquinoline-1-oxide, Meadows-Cook model of alcohol abuse, esophageal squamous cell carcinoma, canonical and noncanonical Wnt signaling, cellular metabolism

## Abstract

Esophageal squamous cell carcinomas (ESCCs) are very common, aggressive tumors, and are often associated with alcohol and tobacco abuse. Because ESCCs exhibit high recurrence rates and are diagnosed at late stages, identification of prognostic and drug targets for prevention and treatment is critical. We used the 4-nitroquinoline-1-oxide (4-NQO) murine model of oral carcinogenesis and the Meadows-Cook model of alcohol abuse to assess changes in the expression of molecular markers during the initial stages of ESCC. Combining these two models, which mimic chronic alcohol and tobacco abuse in humans, we detected increased cellular proliferation (EGFR and Ki67 expression), increased canonical Wnt signaling and downstream elements (β-catenin, FoxM1, and S100a4 protein levels), changes in cellular adhesive properties (reduced E-cadherin in the basal layer of the esophageal epithelium), and increased levels of phosphorylated ERK1/2 and p38. Additionally, we found that treatment with ethanol alone increased the numbers of epithelial cells expressing solute carrier family 2 (facilitated glucose transporter, member 1) (SLC2A1) and carbonic anhydrase IX (CAIX), and increased the phosphorylation of p38. Thus, we identified both 4-NQO- and ethanol-specific targets in the initial stages of esophageal carcinogenesis, which should lead to the development of potential markers and therapeutic targets for human ESCC.

## INTRODUCTION

Cancers of the esophagus, which can be divided into squamous cell carcinomas (ESCCs) and adenocarcinomas, are the eighth most common malignancy worldwide, affecting over 450, 000 people [1]. Esophageal cancers typically are classified as a subgroup of cancers of the upper aerodigestive tract (UADT), which also include malignancies of the oral cavity, pharynx, and larynx [2]. Additionally, esophageal cancers can be grouped with malignancies that affect the digestive system as the fifth most common in terms of new cases and the fourth in terms of estimated deaths in the United States during 2012 [3]. Moreover, ESCC represents one of the top 10 types of cancer-related deaths in males between the ages of 40 and 59 years [3]. Although there have been advances in the diagnosis, operative techniques, and prognosis, the 5 year relative survival rate (5-YRSR) still remains low at 19% [3]. The low 5-YRSR for ESCC can be attributed primarily to diagnosis at an advanced stage, characterized by invasion and metastasis to the lymphatic system and remote organs at the time of diagnosis [4]. Identification of predictive biomarkers to identify and diagnose ESCC at earlier stages, before the observation of frank tumors, would be beneficial for patient survival.

Alcohol and tobacco abuse are major risk factors for the development of ESCC and these two social risk factors may have a synergistic effect on the initiation of ESCC [5, 6]. The odds ratio (OR) for oral cavity cancers of chronic tobacco smokers and alcohol drinkers is 50 to 1, relative to never smokers and drinkers [5]. The International Agency for Research on Cancer (IARC) has determined that chronic alcohol consumption can lead to the development of cancers of the oral cavity, esophagus, and liver [7]. The role of alcohol in the initiation and development of ESCC is closely related to the metabolism of ethanol. Alcohol dehydrogenases (ADHs) oxidize ethanol to acetaldehyde, whose concentrations in alcoholic beverages can reach approximately 200 mM [8]. Acetaldehyde is then metabolized into acetate by aldehyde dehydrogenase-2 (ALDH2) [7]. Acetaldehyde, a genotoxic compound, acts as a carcinogen in humans by inducing mutations, promoting sister chromatid exchange, and interfering with DNA synthesis and repair [7, 9]. The formation of acetaldehyde-derived DNA adducts, such as N^2^-(3-hydroxybutyl)-dG, α-methyl-ϒ-OH-propano-dG, and N^2^-(4-hydroxybutyl)-dG, induces DNA polymerase errors, thus initiating mutations that silence tumor suppressors and/or activate oncogenes [2, 10].

Regarding tobacco abuse, the IARC has determined that cigarette smoke contains over 60 putative carcinogens, with 15 of these, including polycyclic aromatic hydrocarbons (PAHs) and *N*-nitrosamines, confirmed as carcinogenic in humans [11, 12]. The carcinogens in cigarette smoke can be metabolized by cytochrome P450 enzymes into water-soluble forms, generating DNA adducts [13]. For instance, the major adduct of the PAH, Benzo[a]pyrene (BaP), generates G-to-T transversions in DNA. These G-to-T transversions often are preferred sites for the formation of CpG islands, which have been linked to the silencing of tumor suppressor genes [14-16].

To understand the roles of chronic tobacco and alcohol abuse in the initiation of ESCC, we have combined the Meadows-Cook and 4-Nitroquinoline-1-oxide (4-NQO) carcinogenesis models. The Meadows-Cook murine model simulates the effects of chronic alcohol abuse of humans in the oral cavity, the esophagus, and the liver [17]. The 4-NQO murine model of oral and esophageal carcinogenesis in which the carcinogen, 4-NQO, is a surrogate for tobacco [18], has been extensively used to analyze cancers of the oral cavity and esophagus in animal models [19-21]. We and others have used these two models to characterize the molecular effects of chronic alcohol and tobacco abuse in the oral cavity [22-24]. We combined these two models to simulate chronic alcohol and tobacco abuse in humans, to investigate the molecular alterations present during the early stages of ESCC, and to identify ethanol-specific targets in ESCC carcinogenesis. Here we show that changes in the expression of molecular markers involved in cellular proliferation, oncogenic signaling, cellular adhesion, and cellular metabolism occur during the initial stages of ESCC.

## RESULTS

### Histopathological analyses of esophagi from mice subjected to the 4-NQO murine model for oral carcinogenesis and the Meadows-Cook model for chronic alcohol abuse

**Figure 1:**
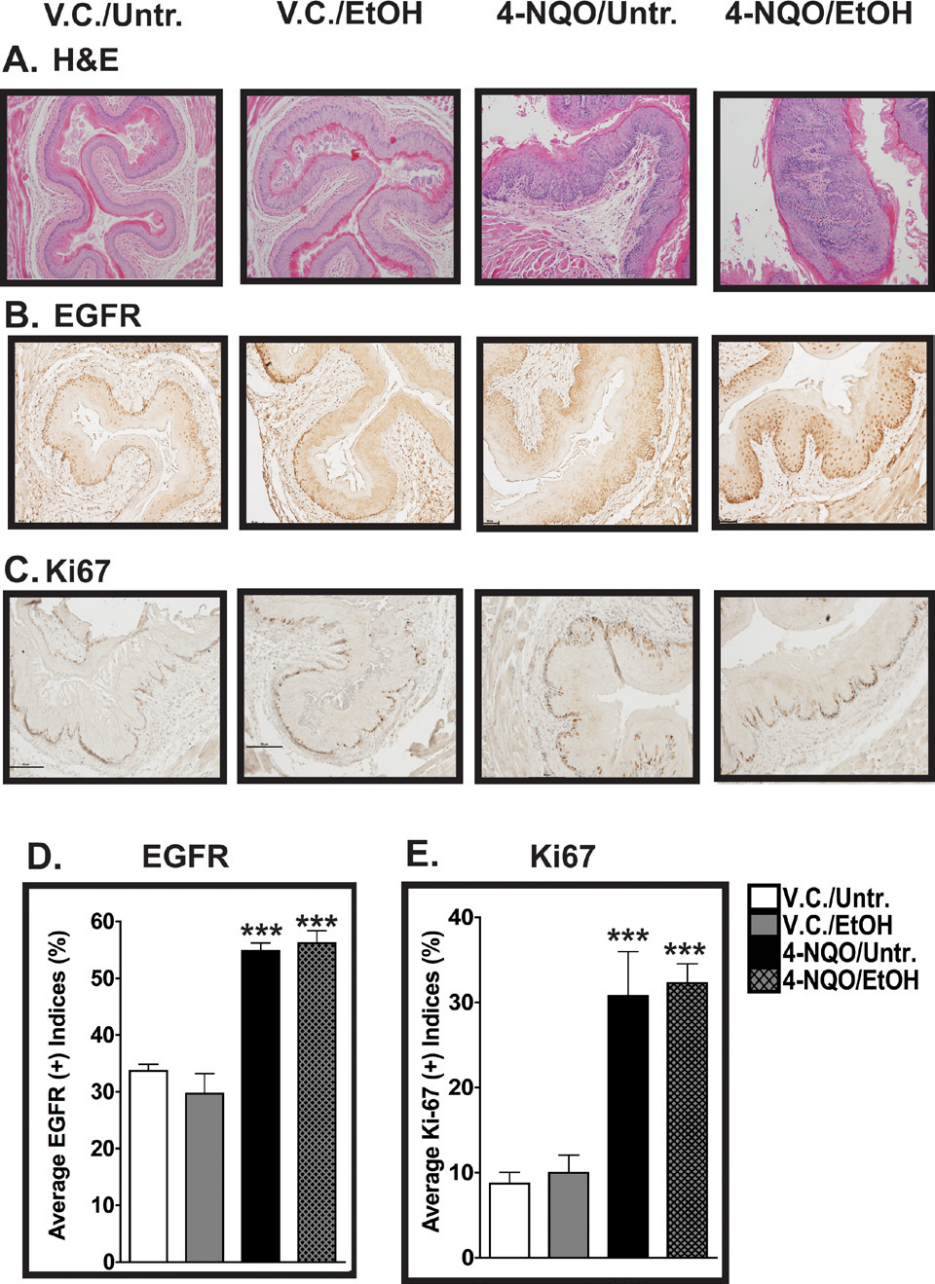
Histopathological and cell proliferation analyses of esophagi of mice treated with 4-NQO and 4-NQO followed by ethanol (A) Representative H&E-stained images of the esophagi were analyzed by a board-certified pathologist to determine changes in the epithelial architecture of the esophagus. (B, C) Immunohistochemistry (IHC) analysis of epidermal growth factor receptor (EGFR) protein (B), a marker of cellular proliferation, and Ki67 (C), a marker of cells in S phase. (D, E) EGFR- and Ki67-positive cells (D and E, respectively) were quantified from the esophagi from the four experimental groups by determining the percentage of stained cells out of the total number of cells in one field from three individual mice. Note that the y-axes are different in D and E. All photos were taken at a 200x magnification with 50 μm scale bars. In D and E, each bar signifies the mean±s.d. of three mice and ANOVA combined with the Tukey post-hoc tests determined statistical significance, where ***, *p*<0.001.

Changes in the histopathology of the epithelia of the esophagi from each experimental group, V.C./Untr. (n=12), V.C./EtOH (n=15), 4-NQO/Untr. (n=15), and 4-NQO/EtOH (n=18) were determined by a board-certified pathologist blinded to the sample identities. Esophagi from the V.C./Untr. and the V.C./EtOH experimental groups did not harbor any pathologic abnormalities in any regions of the tissue (Fig. 1A, and Table 1). In the esophagi of mice treated only with 4-NQO (4-NQO/Untr.), we observed hyperplasia, low-, and high-grade dysplasia – 6.7%, 86.7% and 6.7%, respectively (Table 1 and Fig. 1A). Interestingly, mice treated with 4-NQO and subsequently with ethanol (4-NQO/EtOH) displayed more high-grade dysplasia, 16.7% (Table 1 and Fig. 1A). Although we did not observe any esophageal SCC at these early time points (11 weeks after 4-NQO administration), there were statistically significant levels of low-grade dysplasia in the 4-NQO/Untr. (86.7%, *p*<0.001) and 4-NQO/EtOH (83.3%, *p*<0.001) groups compared to the V.C./Untr. group (Table 1). Also, these histological data suggest an additive effect of ethanol to SCC carcinogenesis induced by 4-NQO. Compared to the V.C./Untr. group, 16.7% (*p*<0.01) of the esophagi analyzed from the 4-NQO/EtOH group displayed significant, high-grade dysplasia (Table 1). These data suggest that ethanol can increase 4-NQO-induced ESCC through modifications in the epithelium of the esophagus.

**Table 1:** High-grade dysplasias in the esophagus are increased by 4-NQO and ethanol

Experimental Group	Number of Esophagi Analyzed	Hyperplasia (%)	Low-Grade Dysplasia (%)	High-Grade Dysplasia (%)
V.C./Untr.	12	0	0	0
V.C./EtOH	15	0	0	0
4-NQO/Untr.	15	1 (6.7)	13 (86.7) ***	1 (6.7)
4-NQO/EtOH	18	0	15 (83.3) ***	3 (16.7) **

Note: ** and *** represent p<0.01 and p<0.001, respectively.

### Treatment with 4-NQO alone and treatment with 4-NQO followed by ethanol increase cellular proliferation in the esophagus

We measured the cellular proliferation of the epithelial cells in the esophagus by quantitative immunohistochemistry (IHC) using antibodies directed against epidermal growth factor receptor (EGFR) and Ki67. EGFR expression has been linked to increased cell proliferation in several types of cancer [25] and Ki67 is a marker that also indicates actively proliferating cells [26]. We found that the percentage of EGFR(+) cells from the V.C./Untr. control group (~32%) was statistically lower than those of the 4-NQO/Untr. (~55%, *p*<0.001) and 4-NQO/EtOH (~56%, *p*<0.001) experimental groups (Fig. 1B, 1D). Compared to the V.C./Untr. group, we did not detect any statistically significant changes in the percentage of EGFR(+) cells in the V.C./EtOH experimental group (~30%) (Fig. 1B, 1D). Also, there were more EGFR(+) cells in the suprabasal layers of the esophagus in the 4-NQO/Untr. and 4-NQO/EtOH experimental groups compared to the V.C./Untr. group (Fig. 1B).

We observed a similar staining profile for Ki67 in the esophagus. The Ki67 staining profiles were as follows: ~9% (V.C./Untr.), ~10% (V.C./EtOH), ~31% (4-NQO/Untr.), and ~32% (4-NQO/EtOH) (Fig. 1C, 1E), with the Ki67 staining percentages of the 4-NQO/Untr. and 4-NQO/EtOH experimental groups compared to the V.C./Untr. group statistically significant (*p*<0.001) (Fig. 1E). We did not observe any statistically significant differences in the V.C./EtOH group compared to the V.C./Untr. group for Ki67 (Fig. 1E). These data indicate that alcohol does not enhance 4NQO-induced carcinogenesis by increasing cell proliferation at this time point.

### Suprabasal expression of E-cadherin is increased in the esophagi of 4-NQO and 4-NQO plus ethanol treated mice

**Figure 2:**
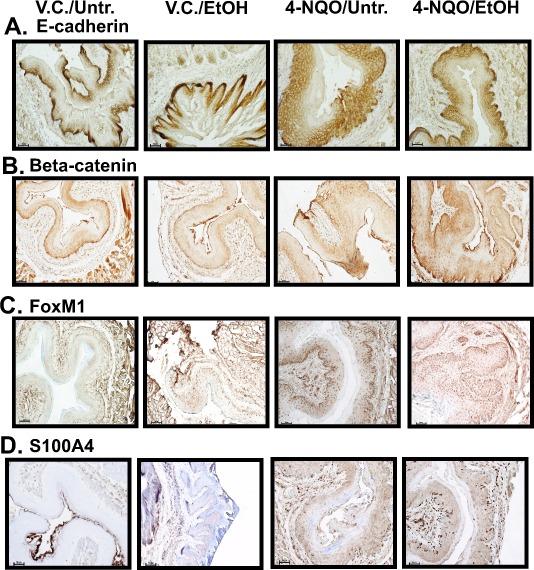
The esophagi of 4-NQO/Untr and 4-NQO/EtOH treated mice display changes in the location of E-cadherin protein and increases in canonical Wnt signaling (A-D) Representative IHC images showing E-cadherin (A), β-catenin (B), Forkhead box M1 (FoxM1) (C), and S100 calcium binding protein A4 (S100A4) (D) positive cells from the V.C./Untr., V.C./EtOH, 4-NQO/Untr., and 4-NQO/EtOH experimental groups. Each image is representative of 3 mice from each experimental group (magnification 200x; 50 μm scale bars).

**Figure 3:**
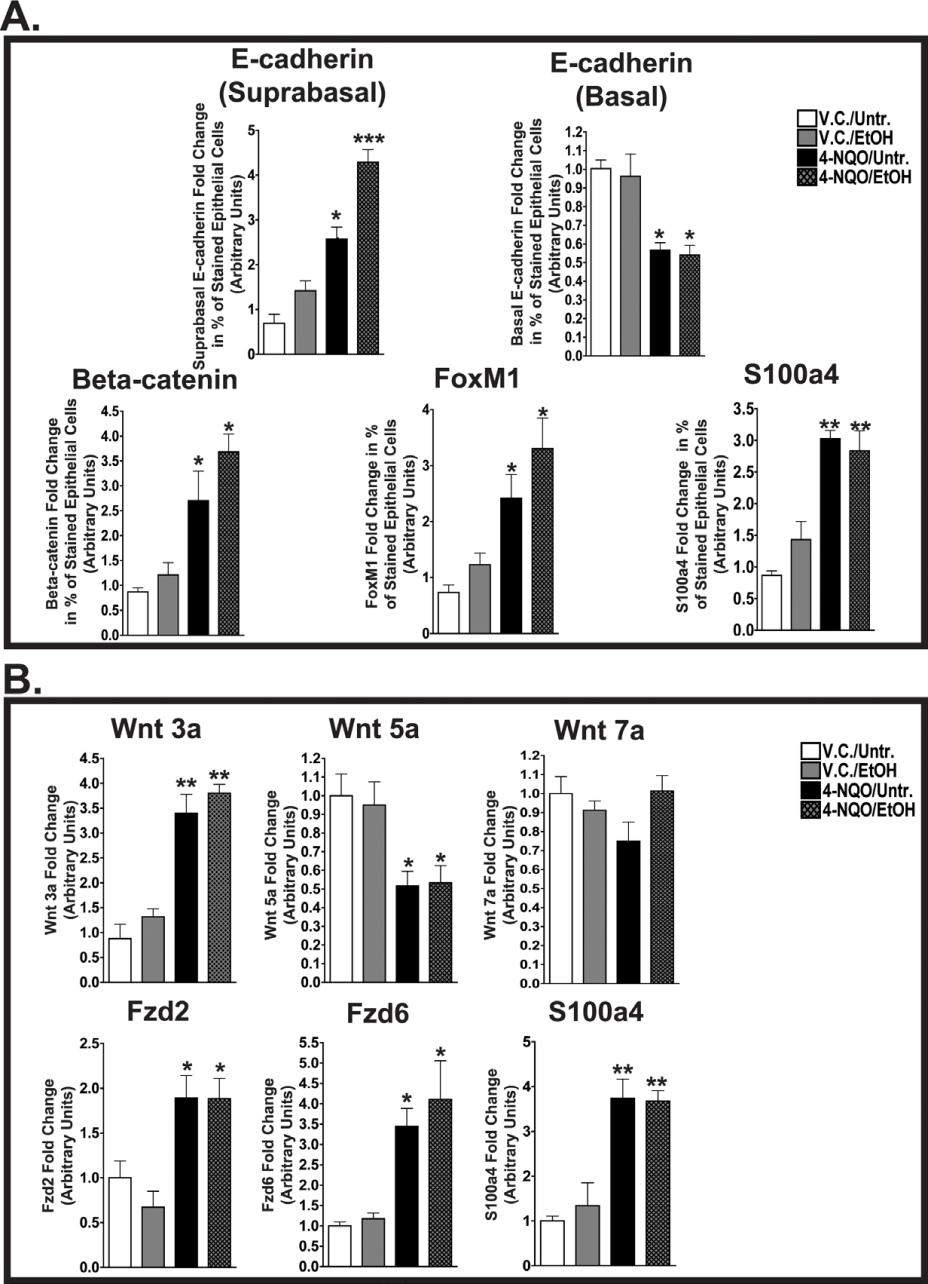
4-NQO and 4-NQO followed by ethanol administration increase E-cadherin in the suprabasal layers, decrease E-cadherin in the basal layer, and increase canonical Wnt signaling during the initiation of ESCC (A) Densitometry analyses, performed by ImageJ analysis software, were used to determine the percentages of cells in the epithelial layer stained by the signaling targets described in Figure 2 - E-cadherin (suprabasal layer expression and basal layer expression), β-catenin, FoxM1, and S100a4. (B) QRT-PCR analysis of transcript levels of a canonical Wnt ligand (Wnt3a), noncanonical Wnt ligands (Wnt5a and Wnt7a), noncanonical Frizzled receptors (Fzd2 and Fzd6), and a canonical downstream target (S100a4). In B, the fold change for each target was determined by normalizing the ratios of the target mRNAs to 36B4 and then to the V.C./Untr. group (set at 1.0). For panels A and B, ANOVA combined with the Tukey post-hoc tests determined statistical significance, where each bar represents mean±s.d. of 3-5 mice and *p<0.05, **p<0.01, and ***p<0.001. Primer pairs for the targets in B can be found in Supplementary Table 1.

Next, we determined the changes in the localization of E-cadherin in the epithelia of the esophagi in the V.C./EtOH, 4-NQO/Untr., and 4-NQO/EtOH experimental groups compared to the V.C./Untr. group (Figs. 2A, and 3A). Reduced E-cadherin expression has been linked to tumorigenesis by inducing invasive and metastatic properties in transformed cells [27, 28]. We detected differences in the percentages of E-cadherin(+) cells within the basal and suprabasal layers of the epithelium (Figs. 2A, 3A). There was an increase of E-cadherin(+) cells in the suprabasal layers of the 4-NQO/Untr. (~2.5 fold, *p*<0.05) and 4-NQO/EtOH (~4 fold, *p*<0.001) groups (Fig. 3A). Also, we detected decreases in the percentages of E-cadherin(+) cells within the basal layer of esophagi compared to the V.C./Untr. (set at 1.0) for the 4-NQO/Untr. (~0.5 fold, *p*<0.05) and 4-NQO/EtOH groups (~0.5, *p*<0.05) (Fig. 3A).

### 4-NQO and 4-NQO followed by ethanol promote changes in Wnt signaling pathways through their ligands, receptors, and downstream targets

The activation of the canonical (β-catenin dependent) Wnt pathway and modifications of the noncanonical (β-catenin independent) Wnt pathways have been associated with the initiation of cellular neoplastic transformation [29]. We observed changes in the protein levels of β-catenin, Forkhead box M1 (FoxM1), and S100 Calcium Binding Protein A4 (S100A4) (Figs 2B-D, 3A). In addition, we investigated changes in the transcript levels of one canonical Wnt ligand (Wnt3a), noncanonical Wnt ligands (Wnt5a and Wnt7a), and Frizzled (Fzd) receptors (Fzd2 and Fzd6) (Fig. 3B). β-catenin staining was observed throughout the entire epithelia (basal and suprabasal layers) in the esophagi of the 4-NQO/Untr. and 4-NQO/EtOH groups (Fig. 2B). This staining pattern was in contrast to the exclusively basal layer expression of β-catenin in the V.C./Untr. and V.C./EtOH groups (Fig. 2B). Compared to the V.C./Untr. group, the average percentages of β-catenin(+) cells in the epithelial layer as fold changes were as follows: V.C./EtOH (~1.1), 4-NQO/Untr. (~3.0, *p*<0.05) and 4-NQO/EtOH (~3.8, *p*<0.05) (Fig. 3A). Also, the 4-NQO/Untr. and 4-NQO/EtOH groups showed a major reduction in phosphorylated β-catenin, which indicates activation of the canonical Wnt signaling pathway (Fig. 6A and B). Compared to the V.C./Untr. group (set at 1.0), the levels of phosphorylated β-catenin were as follows: V.C./EtOH (~0.97), 4-NQO/Untr. (~0.4, *p*<0.001), and 4-NQO/EtOH (~0.45, *p*<0.001) (Fig. 6B).

FoxM1 has been linked to human epithelial stem/progenitor expansion [30] and to advancing elements of the downstream canonical Wnt pathway [31]. Here, we found that administration of 4-NQO (4-NQO/Untr.) and 4-NQO followed by ethanol (4-NQO/EtOH) resulted in increased FoxM1(+) cells in the epithelia in both the basal and suprabasal layers of the esophagus (Fig. 2C). The staining profiles of suprabasal FoxM1(+) cells in the 4-NQO/Untr. and 4-NQO/EtOH groups were similar to the suprabasal expression of β-catenin (Fig. 2 and Fig. 3A). Compared to the V.C./Untr. group (set at 1.0), the percentages of FoxM1(+) cells in the epithelia (as fold changes) were as follows: V.C./EtOH (~1.2), 4-NQO/Untr. (~2.3, *p*<0.05) and 4-NQO/EtOH (~3.1, *p*<0.05) (Fig. 3A).

S100A4, another downstream target of the canonical Wnt signaling pathway, can promote cellular metastasis [32]. Based on our IHC analyses, we found 3.1 (*p*<0.01) and 2.9 (*p*<0.01) fold increases for S100A4(+) cells in the esophagi of 4-NQO/Untr. and 4-NQO/EtOH treated mice, respectively (Figs. 2D, 3A). Also, we observed a similar, statistically significant trend in S100A4 transcript levels in these treatment groups (Fig. 3B).

To understand further the role of canonical and noncanonical Wnt signaling in the initiation of ESCC, we measured the transcript levels of Wnt ligands and Fzd receptors (Fig. 3B). As expected, we detected increases in Wnt3a transcript levels in mice treated with 4-NQO (4-NQO/Untr.) (~3.1 fold, *p*<0.01) and 4-NQO followed by ethanol (4-NQO/EtOH) (~3.5 fold, *p*<0.01; Fig. 3B). Also, we observed a significant decrease in transcripts of Wnt5a, a noncanonical Wnt ligand, in the 4-NQO/Untr. and 4-NQO/EtOH groups (Fig. 3B). However, we did not observe any changes in Wnt7a (another noncanonical Wnt ligand) transcript levels among the four groups (Fig. 3B). Interestingly, there were large increases in the transcript levels of Fzd2 and Fzd6, noncanonical Fzd receptors, in the esophagi of mice from the 4-NQO/Untr. and 4-NQO/EtOH treatment groups (Fig. 3B). These results show that the administration of 4-NQO can modify several markers of the canonical and noncanonical Wnt signaling pathways.

### SLC2A1 (GLUT1) and CAIX positive epithelial cells are increased in the esophagi from mice treated with ethanol and 4-NQO

**Figure 4:**
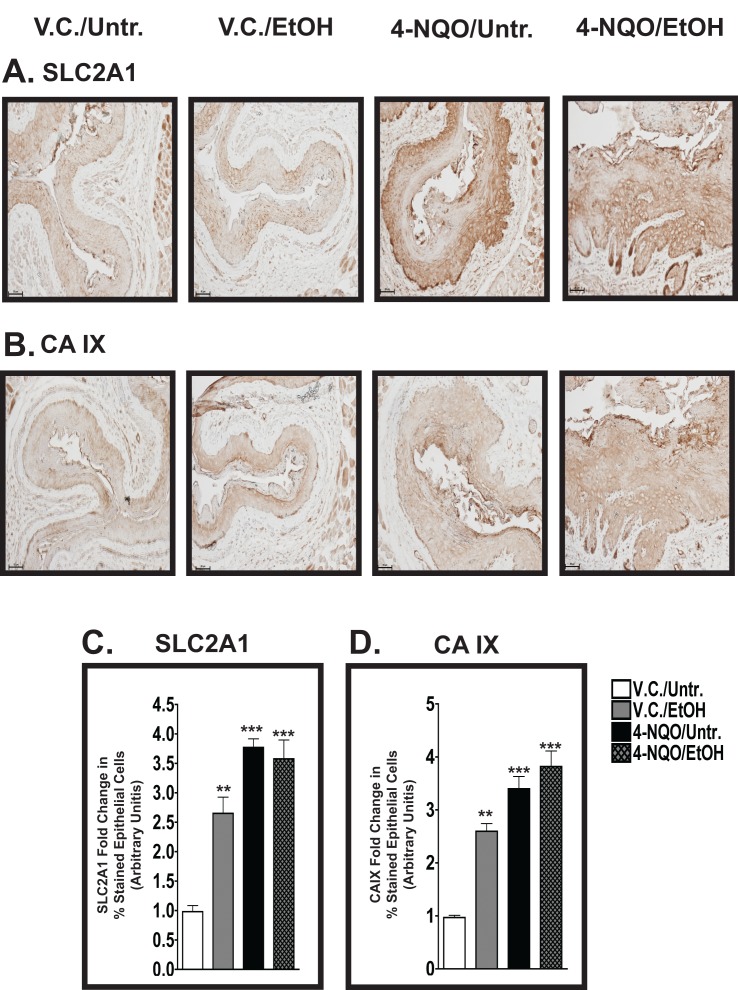
The percentages of cells positive for SLC2A1 and CAIX in the epithelium increase in the esophagus after the administration of either 4-NQO and/or ethanol (A, B) IHC analyses of solute carrier family 2 [facilitated glucose transporter, member 1 [SLC2A1; also known as Glucose transporter 1 (GLUT1); A] and carbonic anhydrase IX (CAIX; B) in the esophagi of V.C./Untr., V.C./EtOH, 4-NQO/Untr., and 4-NQO/EtOH treatment groups. These representative images were taken at 200x magnification with 50 μm scale bars. (C, D) Semiquantitative staining analysis determines that the V.C./EtOH, 4-NQO/Untr., and 4-NQO/EtOH treatment groups have significant leves of SLC2A1(+) cells (C) and CAIX(+) cells (D).. Data from three mice from each experimental group are expressed as mean±s.d. and were subjected to ANOVA and post-hoc statistical tests, where *, *p*<0.05; **, *p*<0.01; and ***, *p*<0.001.

Solute carrier family 2A (facilitated glucose transporter, member 1) [SLC2A1; also known as glucose transporter 1 (GLUT1)] protein is increased in kidney, oral cavity, breast, and prostate cancers [33]. From our IHC analyses, we detected increases in SLC2A1(+) cells in the V.C./EtOH (~2.8 fold, *p*<0.01), 4-NQO/Untr. (~3.5 fold, *p*<0.001) and 4-NQO/EtOH (~3.3 fold, *p*<0.001) groups compared to the V.C./Untr. group (set at 1.0) (Fig. 4A, 4C).

Also, the acidification of the tumor microenvironment by carbonic anhydrases (CAs) has been implicated in tumor initiation and progression via activation of angiogenic factors, increased cellular migration, and reduced cell-cell adhesion [34]. Similar to the SLC2A1 staining profile, we observed CAIX(+) cells in both basal and suprabasal layers in the epithelia of the esophagi in all four experimental groups (Fig. 4B, 4D). Based on the average percentages of cells stained, the fold changes in CAIX staining in the esophagi were as follows: ~2.5 (V.C./EtOH; *p*<0.01), ~3.2 (4-NQO/Untr.; *p*<0.001), and ~4.7 (4-NQO/EtOH; *p*<0.001) compared to V.C./Untr., set at 1.0 (Fig. 4D). These increases in SLC2A1 (Fig. 4A, 4C) and CAIX (Fig. 4B, 4D) in the V.C./EtOH group could potentially be employed to detect the early stages of ESCC.

### β-Catenin, FoxM1, and SLC2A1 protein levels are similarly increased in human ESCC

**Figure 5:**
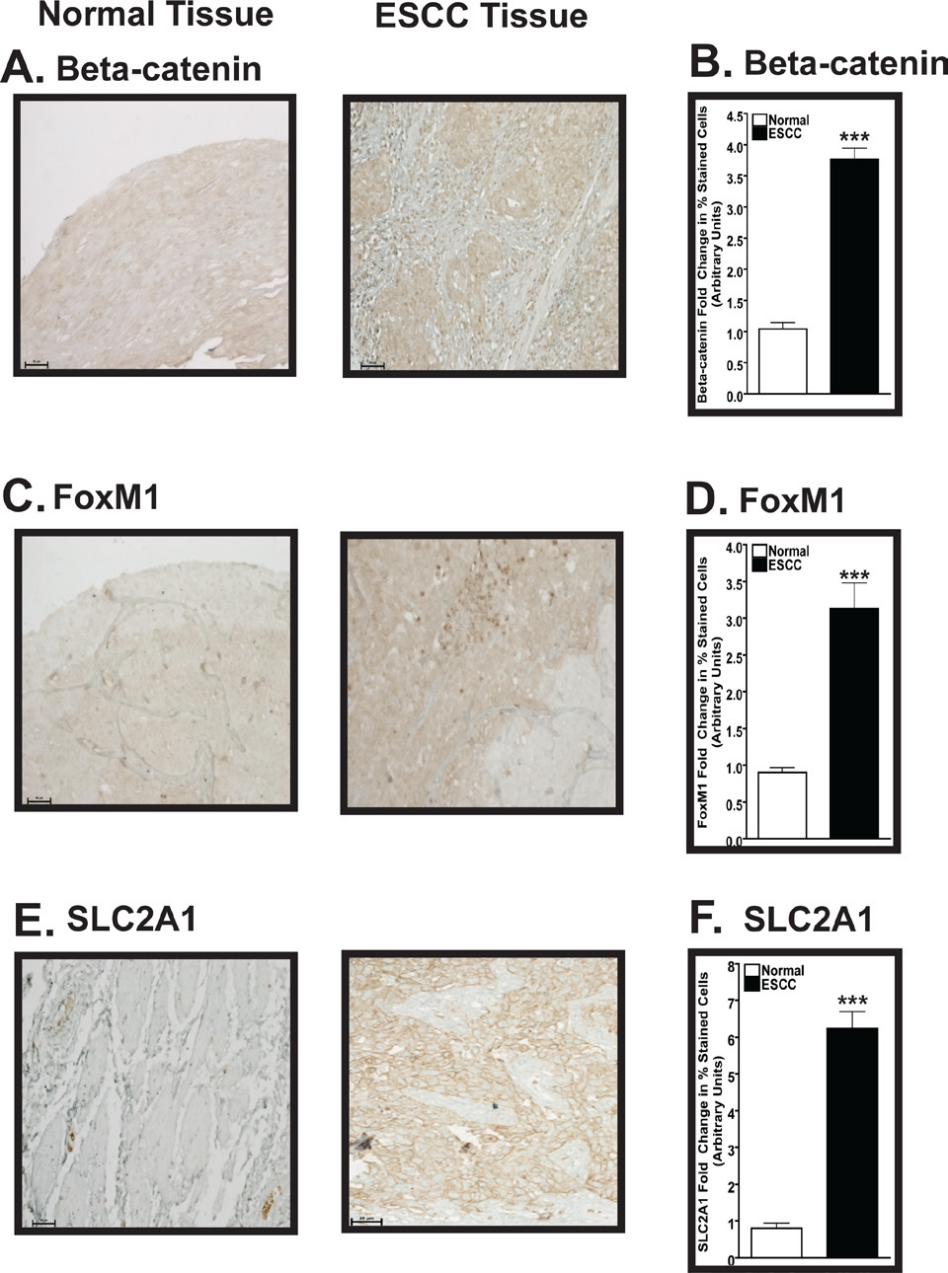
Expression of β-catenin, FoxM1, and SLC2A1 is increased in human ESCC compared to normal esophageal tissue (A, C, E) Representative IHC analysis demonstrating the levels of β-catenin (A), FoxM1 (C), and SLC2A1 (E) in normal (left panel) and ESCC (right panel) tissues. These representative images were taken at 200x magnification with 50 μm scale bars. (B, D, F) Semiquantitative IHC analysis determines increased β-catenin (B), FoxM1 (D), and SLC2A1 (F) protein levels in human ESCC versus normal esophageal tissue. For the semiquantitative IHC analysis, each bar signifies the mean±s.d. of three patients and ANOVA combined with the Tukey post-hoc tests determined statistical significance, where ***, *p*<0.001. Note that the y-axes are different for B, D, and F.

Using a human ESCC tissue microarray, we investigated the protein levels of β-catenin, FoxM1, and SLC2A1 in the esophagi of human patients diagnosed with ESCC (Fig. 5). Based on immunohistochemical (IHC) analysis of the human ESCC tissue microarrays, the average fold increases for these three targets in the malignant tissues were as follows: ~3.2 (β-catenin; *p*<0.001), ~3.1 (FoxM1; *p*<0.001), and 6.3 (SLC2A1; *p*<0.001), compared to normal tissue (set at 1.0) (Fig. 5B, 5D, 5F). The malignant tissue sections from the clinical microarrays were characterized as either moderately-differentiated or poorly-differentiated ESCC (Supplementary Table 2).

### The administration of 4-NQO and 4-NQO followed by ethanol increases phosphorylated ERK 1/2 levels and both 4-NQO and ethanol treatments increase the phosphorylation of p38

**Figure 6:**
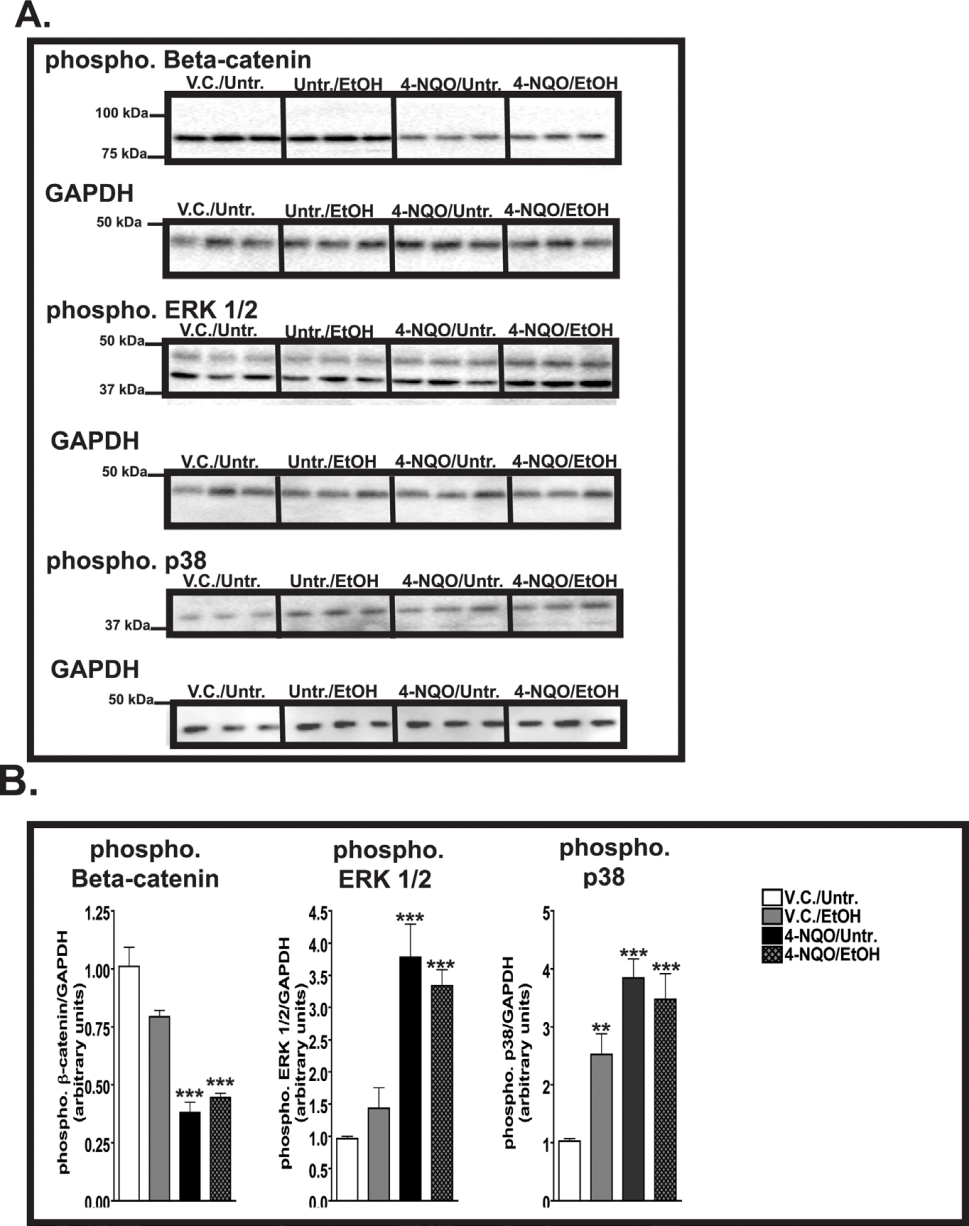
β-catenin and ERK 1/2 and p38 are activated by ethanol, 4-NQO, and 4-NQO followed by ethanol treatment (A) Representative Western blots displaying 30 μg of total protein extracted from the esophagi of 3 mice from each experimental group showing the levels of phosphorylated β-catenin, phosphorylated ERK 1/2, and phosphorylated p38. The GAPDH blots, which represent loading controls, are located directly below the corresponding phosphorylated targets. (B) Quantitative analysis of phosphorylated levels of β-catenin, ERK 1/2, and p38. The fold change for each target was determined by normalizing the ratios of the target phosphorylated proteins levels to GAPDH protein levels and then to the V.C./Untr. group. Statistical significance was determined by repeating the Western blotting analyses three times with the same samples to ensure the reproducibility of the blots, where **p<0.01 and ***p<0.001. Note that the y-axes are different for each graph in B.

The development and progression of the early stages of tumorigenesis can be influenced by the phosphorylation activities in the mitogen activated protein kinase (MAPK) pathways [35]. We investigated the changes in the phosphorylation levels of ERK 1/2 and p38. Compared to the V.C./Untr. group (set at 1.0), we detected 3.8 (*p*<0.001) and 3.2 (*p*<0.001) fold increases in the levels of phosphorylated ERK 1/2 in the esophagi of mice from the 4-NQO/Untr. and 4-NQO/EtOH groups, respectively (Fig. 6A, 6B). In addition, we showed that the phosphorylation of p38 may be a target of tumorigenesis induced by 4-NQO and potentially by ethanol. Compared to the V.C./Untr. group (set at 1.0), the levels of phosphorylated p38 were as follows: V.C./EtOH, ~2.3 (*p*<0.01); 4-NQO/Untr., ~3.8 (*p*<0.001); and 4-NQO/EtOH, ~3.5 (*p*<0.001) (Fig. 6A, 6B).

## DISCUSSION

### Chronic 4-NQO and 4-NQO/EtOH treatments induce changes in cell proliferation in the esophagus

By combining the 4-NQO carcinogenesis and Meadows-Cook models for chronic alcohol abuse we delineated changes in various signaling pathways during the initiation of ESCC. We harvested esophageal tissue from mice at only 11 weeks after the termination of 10 weeks of 4-NQO administration so that we could analyze the early events in esophageal carcinogenesis. We show that the 4-NQO/Untr. and 4-NQO/EtOH treatments induce major changes in the architecture of the epithelium (Table 1 and Fig. 1A). Compared to the V.C./Untr. group, we measured increased levels of low-grade dysplasia in the 4-NQO/Untr. and 4-NQO/EtOH groups and increased levels of high-grade dysplasia in the 4-NQO/EtOH group (Table 1). Thus, ethanol enhanced the early steps (hyperplasia and dysplasia) of 4-NQO-induced carcinogenesis in the esophagus.

To analyze cell proliferation during the initiation of ESCC, we used quantitative IHC to measure the staining of EGFR and Ki67 in the esophagus (Fig. 1B-E). Both EGFR [25] and Ki67 [26] have been used extensively in IHC analyses as markers to characterize proliferation in cancers of the oral cavity and esophagus in both humans and mice. In both the V.C./Untr. and V.C./EtOH groups, EGFR(+) (Fig. 1B, 1D) and Ki67(+) (Fig. 1C, 1E) cells were limited to the basal layer. In the 4-NQO/Untr. and 4-NQO/EtOH groups, in contrast, we detected suprabasal EGFR staining (Fig. 1B). We previously reported increases in these markers in the suprabasal layers of the tongues of mice in both the 4-NQO/Untr. and 4-NQO/EtOH experimental groups [24]. Thus, ESCC induced by 4-NQO administration could involve horizontal expansion of epithelial basal stem cells, which has been demonstrated in the tongue [23] and the skin [36].

### 4-NQO and 4-NQO followed by ethanol treatments modify E-cadherin expression in the esophagus

In normal tissues E-cadherin mediates cell-cell adhesion and functions as a tumor suppressor; however, its deregulation can initiate epithelial-to-mesenchymal transition (EMT) and tumor metastasis [37]. Often, reduced E-cadherin expression occurs at the genetic level through mutations and gene hypermethylation [38]. Here, we detected approximately a 50% reduction in the number of epithelial basal cells that are E-cadherin(+) in the 4-NQO/Untr. and 4-NQO/EtOH groups compared to the V.C./Untr. group (Figs. 2A, 3A). In the 4-NQO/Untr. and 4-NQO/EtOH groups we detected increased numbers of E-cadherin stained cells in the suprabasal layers (Figs. 2A, 3A).

### Administration of 4-NQO activates the canonical Wnt signaling pathway during the initial stages of ESCC

Increased activity of the canonical Wnt signaling pathway, indicated by the nuclear localization of β-catenin and its interaction with Wnt responsive elements, has been characterized in ESCC via increased expression of downstream factors involved in tumorigenesis, such as Cyclin D1 and c-Jun [39]. We measured increased numbers of murine esophageal cells that were β-catenin, FoxM1, and S100A4 positive by IHC (Figs. 2, 3), and we found reduced levels of phosphorylated β-catenin protein by Western blotting (Fig. 6), similar to what we demonstrated in the tongue [24]. Additionally, we detected increased numbers of cells that express β-catenin(+) (Fig. 5A, B) and FoxM1(+) cells (Fig. 5C, D) in human patients diagnosed with ESCC.

Increased FoxM1 expression has been implicated in many types of cancers, including HNSCC and ESCC [40]. More specifically, high expression of FoxM1 induces changes in DNA methylation and epigenetic remodeling programs to maintain stem/progenitor cell renewal and to antagonize pathways inducing differentiation [41, 42]. Modifications in the proliferative nature of stem/progenitor cells have been implicated in the initiation and recurrence of several cancers, including HNSCC and OSCC [43, 44]. FoxM1 also directly interacts with β-catenin during glioma tumorigenesis to induce nuclear localization of β-catenin, a clear indication of canonical Wnt signaling activation [31]. Moreover, FoxM1 has been linked to the development of chemotherapeutic drug resistance. Additionally, Oncomine microarray analyses show that FoxM1, along with other Forkhead box transcript levels (e.g. FoxK1 and FoxK2) are greatly increased in human ESCC (Supplementary Table 3). Further analysis of the Forkhead box transcript and protein levels could identify additional roles of this family of transcription factors in esophageal tumorigenesis.

Metastasis is a major characteristic of late stage tumorigenesis in ESCC and HNSCC. In nontransformed cells, the S100A subfamily of proteins influences calcium homeostasis, which controls cell survival, differentiation, and metabolism [45]. In the esophagus, S100A4 may contribute to the metastatic stage by inducing the phosphorylation of Akt, mTOR, and p70S6K, and by interactions with myosin IIA and various matrix metalloproteinases (MMPs) [46]. Although we did not observe metastasis at the time point assayed in our model, we did detect increased numbers of cells expressing S100A4, both at the protein (Figs 2D, 3A) and mRNA levels (Fig. 3B), in the esophagi of the 4-NQO/Untr. and 4-NQO/EtOH treated mice.

Activation of the canonical Wnt signaling pathway can induce neoplastic transformation [47]. However, the definitive role for the noncanonical Wnt signaling pathway in cancer remains controversial. Reports have demonstrated both over- and underexpression in Wnt5a transcripts, a key noncanonical Wnt ligand, in tumorigenesis [48]. We detected reduced levels of Wnt5a transcripts, but increased Fzd2 and Fzd6 noncanonical Wnt receptors, in the 4-NQO/Untr. and 4-NQO/EtOH groups compared to the V.C./Untr. group (Fig. 3B). Also, we did not detect any changes in the transcript levels of Wnt7a (Fig. 3B), another noncanonical Wnt ligand. Oncomine data from human ESCC specimens show that Wnt5a (Supplementary Table 4) mRNA is reduced and Fzd2 and Fzd6 (Supplementary Table 3) transcripts are increased, similar to our data in this murine model. The noncanonical Wnt signaling pathway may prevent early transformation of cells at the expense of canonical Wnt signaling; however, the noncanonical pathway can enhance cellular invasiveness after transformation [49]. Additional investigations will have to be conducted to delineate the role of noncanonical Wnt signaling in carcinogen-induced ESCC.

### Ethanol increases SLC2A1- and CAIX-positive epithelial cells in the esophagus

In many types of the cancer, hypoxia (characterized by a low concentration of oxygen) can induce changes in the tumor microenvironment that switch cellular metabolism from oxidative phosphorylation to glycolysis, thus increasing glycogen synthesis and the use of glutamine instead of glucose for energy production [50]. In addition, hypoxia is often associated with low survival rates, poor responses to chemotherapy, and higher resistance to anticancer drugs in many cancers [50]. Although there is substantial information regarding SLC2A1 (also known as GLUT1) and CAIX expression in HNSCC and other malignancies [51, 52], the information is more limited for animal models of ESCC.

We detected increased percentages of SLC2A1(+) cells in the esophagi of mice after the administration of ethanol, 4-NQO, and 4-NQO followed by ethanol (Fig. 4A, 4C). Even though there are very few reports linking elevations in SLC2A1 to ESCC, published microarray data show that SLC2A1 is in the top 10% of overexpressed transcripts in two separate data sets generated from ESCC patients (Supplementary Table 5). Transcripts of other SLC family members involved in cell metabolism, eg. SLC2A2 (GLUT2), SLC2A3 (GLUT3), and SLC16A1 (monocarboxylic acid transporter 1; MCT), are also overexpressed in ESCC patients (Supplementary Table 5). However, the levels and duration of alcohol and/or tobacco consumption by these patients were not reported in these data sets. Finally, a similar increase in SLC2A1 protein levels in the esophagi of 4-NQO/Untr. and 4-NQO/EtOH treated mice also was observed in the malignant tissue from a human ESCC tissue microarray (Fig. 5E, 5F).

We also identified CAIX as a possible target of alcohol exposure in ESCC, as we measured increases in the percentages of CAIX(+) epithelial cells expressing CAIX protein in the esophagi of mice in the V.C./EtOH, 4-NQO/Untr., and 4-NQO/EtOH groups compared to the V.C./Untr. group (Fig. 4B, 4D). These data implicate glucose metabolism (i.e. SLC2A1) and carbonic anhydrases (i.e. CAIX) as potential biomarkers for the contribution of chronic alcohol consumption to the initial stages of ESCC.

### The administration of 4-NQO followed by ethanol is associated with induction of MAPK pathways through the phosphorylation of ERK 1/2 and p38

The members of the mitogen-activated protein kinase (MAPK) family can mediate various cellular functions and responses that are induced by growth factors, hormones, and cellular stress. Aberrant regulation of MAPK signaling is seen in many cancer types, including ESCC. The four major MAPK pathways include the extracellular signal-regulated kinase (ERK), Big MAP kinase-1 (BMK1), p38, and c-Jun N terminal kinase (JNK), where the ERK and BMK1 pathways respond to various growth factors and hormone signals and the JNK and p38 pathways respond to extracellular stress signals [53]. Here, we detected higher levels of phosphorylated ERK 1/2 after the administration of 4-NQO and 4-NQO/EtOH (Fig. 6A, 6B), similar to what we and others reported in the oral cavity and esophagus [24, 54-56].

We measured increased levels of phosphorylated p38 in the V.C./EtOH group compared to the V.C./Untr. group (Fig. 6A, 6B). We also observed increased levels of total p38 in the oral cavities of the V.C./EtOH group [24]. The activation of p38 through phosphorylation on Thr180 and Tyr182 residues has been associated with the angiogenic, invasive, and migratory properties of ESCC [57]. p38 contributes to immune and inflammatory responses by causing the release of tumor-related cytokines and chemokines such as interleukin-6 (IL-6), platelet-derived growth factor (PDGF), and vascular endothelial growth factor (VEGF) in ESCC [57, 58]. Also, activation of the p38 pathway may influence the activation of the canonical Wnt signaling pathway in transformed cell lines and embryonic stem cells [59, 60]. More specifically, p38 inhibits the phosphorylating capacity of GSK-3β, which participates in the degradation of β-catenin by phosphorylating the Ser33/37 and Thr42 residues of β-catenin [60]. Our study shows that the activation of the p38 MAPK pathway, possibly through a response to extracellular stress, may be an ethanol-specific effect in the initiation of ESCC.

## CONCLUDING REMARKS

We have combined the 4-NQO model of oral carcinogenesis and the Meadows-Cook model of chronic alcohol abuse to investigate the molecular changes associated with the initial stages of esophageal carcinogenesis. In both the tongue [23, 24] and in the esophagus, we saw increased cell proliferation, increased expression of canonical Wnt signaling markers, and changes in the expression of cell-cell adhesion molecules. Additionally, we detected similar expression patterns of makers involved in the canonical Wnt signaling pathway and glycolysis in malignant esophageal tissue derived from human ESCC patients. Because of the similarities of the esophageal epithelial tissue between humans and mice the combination of the 4-NQO and Meadows-Cook models serves as an excellent model to determine prognostic markers for early ESCC and to analyze potential drugs for prevention or treatment of ESCC.

## MATERIALS AND METHODS

### Animals and treatments

As described in [24], eight week old female wild-type C57BL/6 mice (Jackson Laboratory, Bar Harbor, ME) were subjected to the combined 4-NQO murine model of oral carcinogenesis and Meadows-Cook model of chronic ethanol abuse. Four experimental groups were randomized as follows: Vehicle Control/Untreated (V.C./Untr.), Vehicle Control/Ethanol (V.C./EtOH), 4-NQO/Untreated (4-NQO/Untr.), and 4-NQO/Ethanol (4-NQO/EtOH). Depending on the experimental group, the mice were administered propylene glycol (V.C.) or 100 μg/ml 4-NQO (Cat# N8141, Sigma, St. Louis, MO) for 10 weeks [18, 23], and then given either normal water (Untr.) or water containing 20% (w:v) ethanol (EtOH) for 10 weeks after an one week ethanol acclimation period [24]. This experiment was performed twice, with experiments one and two utilizing 5 and 15 mice per treatment group, respectively.

### Esophagus tissue processing

Hematoxylin and eosin (H&E)-stained, paraffin-embedded cross sections of the esophagi were analyzed by a trained pathologist (T.S.) blinded to the four experimental groups. The sections of the esophagi from the H&E-stained slides were classified as normal, hyperplasic, dysplastic (low- or high-grade), or squamous cell carcinoma (SCC).

### RNA isolation, reverse transcription, and QRT-PCR

Esophagi were homogenized in TRIzol (Cat# 155596-026, Life Technologies, Norwalk, CT) and total RNA extraction was performed as described by the manufacturer. QRT-PCR was performed after reverse transcription of the total RNA (1 μg). Primer pairs used for the QRT-PCR can be found in Supplementary Table 1.

### Immunohistochemical (IHC) analysis

Protein targets were detected using paraffin-embedded sections of murine esophagi, as described [24]. Additionally, IHC analysis was performed using a commercially available ESCC tissue microarray (Cat# BC02021, U.S. Biomax, Inc., Rockville, MD), which contained normal and malignant tissue samples. Patient information (age, sex, pathology diagnosis, and grade) can be found in Supplemental Table 2 and corresponding H&E stained images can be found at http://www.biomax.us/tissue-arrays/Esophagus/BC2021. After deparaffinization, rehydration, and citrate-based antigen unmasking, the slides were blocked with mouse anti-IgG blocking solution (Cat# MKB-2213, Vector Laboratories, Burlingame, CA), rat anti-IgG blocking solution (Cat# CTS005, R&D Systems, Minneapolis, MN), or PBS containing 10% goat serum and 0.1% Tween (for rabbit primary antibodies). Following primary mouse, rat, or rabbit primary antibody incubation, the slides were incubated with secondary antibodies from the M.O.M (Cat# MKB-2213, Vector Laboratories), Rat Cell & Tissue Staining HRP-Dab (Cat# CTS005, R&D Systems), or SuperPicture HRP Polymer Conjugate (Cat# 87-8963, Life Technologies) kits, respectively. Details regarding the antibodies used can be found in the Supplementary Materials and Methods.

### Semiquantitative IHC analysis

Formalin-fixed, paraffin-embedded esophageal sections (3-5 from each experimental group) were photographed at 200x magnification. Using ImageJ 1.48v (http://rsp.info.nih.gov/ij), the photographs were resolved into separate RGB channels, where the green channel was used for analysis. Within each photo, only the epithelial layer was selected for analysis by setting the threshold levels to a point were DAB(+) cells were selected. Then ImageJ calculated the total area of the DAB(+) cells compared to the total area of the epithelial layer. The results are shown as the fold changes in percent of the epithelial layer positive for the stain by comparing to the photographs taken from the V.C./Untr. experimental groups, where the percent positive was set at 1.0.

### Western blotting analysis

Total protein lysates (30 μg) were extracted from the esophagi of three mice from each experimental group and Western blotting was performed three times as described in [24]. Details regarding the antibodies used for Western blotting can be found in the Supplementary Materials and Methods.

### Oncomine gene expression data analysis

The top 10% under and overexpressed transcripts of various genes in human ESCC-derived tissue were obtained from the Oncomine Cancer Microarray database analysis (http://www.oncomine.org). The following data sets were used: Hu Esophagus (RNA data set comprised of 12, 603 genes from 34 patients) and Su Esophagus 2 (RNA data set comprised of 17, 779 genes from 106 patients).

### Statistical analyses

The Fisher's exact probability test was used to determine significance among the distribution of dysplasia in each treatment group. Statistical significance for IHC, QRT-PCR, and Western blotting was determined by using analysis of variation (ANOVA) and the Tukey posthoc tests (Prism 4, GraphPad Software, Inc.). ANOVA and Tukey posthoc tests were performed by comparing the V.C./Untr. control group to the V.C./EtOH, 4-NQO/Untr., and 4-NQO/EtOH experimental groups, where statistical significance was set at *p*<0.05. For these analyses, the fold changes in protein levels were assessed by normalizing the means of each group to the V.C./Untr. group. Note, only the average staining intensities were used to determine statistical significance for the IHC analyses.

## SUPPLEMENTARY MATERIALS AND METHODS TABLES


