# Supplementary material for: Initiation of esophageal squamous cell carcinoma (ESCC) in a murine 4-nitroquinoline-1-oxide and alcohol carcinogenesis model
Source: Oncotarget. 2015 Jan 21;6(8):6040–52. doi: 10.18632/oncotarget.3339 (PMC4467420; doi:10.18632/oncotarget.3339)
Supplement: Supplementary file 1 [file oncotarget-06-6040-s001.pdf]

Initiation of esophageal squamous cell carcinoma (ESCC) in a murine 4-nitroquinoline-1-oxide and alcohol carcinogenesis model

Supplementary Materials and Methods:

Animals and Treatments

The esophageal samples were obtained from two separate experiments in which experiment 1 had a sample size of 5 for each experimental group and experiment 2 had a sample size of 15 for each experimental group. During the ethanol treatment, the body weights of two randomly selected mice from each cage were monitored weekly. Also, the food intake and the water/ethanol intake from each cage were measured weekly [24]. All procedures were performed in accordance with the NIH and approved by the WCMC Institutional Animal Use and Care Committee.

Immunohistochemical analysis

Following blocking, the sections were incubated with the following antibodies: mouse monoclonal anti-E-cadherin (1:2000; Cat# ab53033, Abcam, Cambridge, MA), rat polyclonal anti-Ki67 (1:300; Cat# M7249, Dako, Carpentaria, CA), rabbit polyclonal anti-β-catenin (1:500; Cat# 6302, Abcam), rabbit anti-Glucose Transport 1 (Glut 1) (1:400; Cat# ab14683, Abcam), rabbit polyclonal anti-carbonic anhydrase IX (1:500; Cat# sc256000, Santa Cruz Biotechnology, Inc., Santa Cruz, CA), rabbit polyclonal anti-IL6 (1:100; Cat# ab6672, Abcam), rabbit polyclonal anti-FoxM1 (C-20) (1:1000; Cat# sc502, Santa Cruz Biotechnology, Inc.), and rabbit polyclonal anti-S100A4 (1:500, Cat# A5114, Dako).

Western blotting analysis

The following antibodies were used for the Western blotting analyses: rabbit polyclonal anti-phospho (Ser33/37/Thr42) β-catenin (1:1000; Cat# 53050; Abcam), rabbit polyclonal anti-phospho p44/42 MAPK (ERK1/2) (Thr202/Tyr204) (1:1000; Cat# 9101; Cell Signaling Technology), rabbit polyclonal anti-phospho

p38 (MAPK) (Thr180/Tyr182) (1:500; Cat# 9211; Cell Signaling Technology), and mouse monoclonal anti-glyceraldehyde 3-phosphate dehydrogenase (GAPDH) (1:2000; Cat# 9484; Abcam).

Supplementary Table 1: QRT-PCR Primer Pairs

Gene	Forward Primer (5'->3')	Reverse Primer (5'->3')	Amplicon size (bp)
36B4	AGAACAACCCAGCTCTGGAGAA	ACACCCTCCAGAAAGCGAGAGT	484
Wnt3a	GGCGCTGCTTCTAATGGAG	TGGGCATGATCTCGATGTAA	218
Wnt5a	TGAACAGTTGCATTTATA	GGACGATACTCCAGGCAGAG	179
Wnt7a	GACAAATACAACGAGGCCGT	GGCTGTCTTATTGCAGGCTC	247
Fzd2	ATCTGGAAACCTCCCAATCC	CGTTTTGTTGCCCATTCTCT	184
Fzd6	AGCCACCACACTCAGCTTTT	CACTCTTCCTGCCCAACTC	190
S100a4	GGAGCTCAAGGAGTTGTTGC	CACTTCGCAGTCTTTGTTGG	115

Supplementary Table 2: Human ESCC Tissue Microarray Pathological Specifications.

Patient #	Sex	Age	Pathology Diagnosis	Grade*	Targets
1	M	56	SCC	III	β-catenin, SLC2A1
1	M	56	Normal	--	β-catenin, SLC2A1
2	M	61	SCC	III	β-catenin, SLC2A1
2	M	61	Normal	--	β-catenin, SLC2A1
3	M	62	SCC	II	β-catenin, SLC2A1
3	M	62	Normal	--	β-catenin, SLC2A1
4	F	55	SCC	III	FoxM1
4	F	55	Normal	--	FoxM1
5	M	56	SCC	II	FoxM1
5	M	56	Normal	--	FoxM1
6	M	60	SCC	III	FoxM1
6	M	60	Normal	--	FoxM1

* Grade II moderately-differentiated ESCC.
* Grade III poorly-differentiated ESCC.

Supplementary Table 3: Selected Canonical and Noncanonical Wnt Signaling Overexpression in Human ESCC.

Gene Name	Gene Alias	Top 10% Overexpression Study
Wingless-type MMTV integration site family member 2	WNT2	Hu
Frizzled homolog 1 (Drosophila)	FZD1	Su 2
Frizzled homolog 2 (Drosophila)	FZD2	Hu, Su 2
Frizzled homolog 3 (Drosophila)	FZD3	Hu
Frizzled homolog 6 (Drosophila)	FZD6	Hu, Su 2
Forkhead box K1	FOXK1	Su 2
Forkhead box K2	FOXK2	Hu, Su 2
Forkhead box M1	FOXM1	Hu, Su 2

Supplementary Table 4: Selected Canonical and Noncanonical Wnt Signaling Underexpression in Human ESCC.

Gene	Gene Alias	Top 10% Underexpression Study
wingless-type MMTV integration site family, member 4	WNT4	Hu
wingless-type MMTV integration site family, member 5A	WNT5A	Hu
wingless-type MMTV integration site family, member 5B	WNT5B	Hu
frizzled homolog 4 (Drosophila)	FZD4	Su 2
Forkhead box N1	FOXN1	Su 2
Forkhead box O1	FOXO1	Su 2
Forkhead box O3	FOXO3	Su 2
Forkhead box O3B pseudogene	FOXO3B	Su 2

Supplementary Table 5: Selected Solute Carrier Family Overexpression in Human ESCC.

Gene	Gene Alias(es)	Top 10% Overexpression Study
Solute carrier family 2 (facilitated glucose transporter), member 1	SLC2A1, GLUT1	Hu, Su 2
Solute carrier family 2 (facilitated glucose transporter), member 3	SLC2A3, GLUT3	Hu
Solute carrier family 16, member 1 (monocarboxylic acid)	SLC16A1, MCT	Hu, Su 2
